# Assessing Barriers to use of the Specific Carbohydrate Diet in Pediatric Inflammatory Bowel Disease: A Qualitative Study

**DOI:** 10.1097/PG9.0000000000000239

**Published:** 2022-07-29

**Authors:** Naomi R.M. Schwartz, Savannah R. McNichol, Beth Devine, Amanda I. Phipps, Joshua A. Roth, David L. Suskind

**Affiliations:** From the * CHOICE Institute, Department of Pharmacy, School of Pharmacy, University of Washington, Seattle, WA; † School of Public Health, Oregon Health & Science University, Portland, OR; ‡ Department of Epidemiology, School of Public Health, University of Washington, Seattle, WA; § Genentech, Inc., San Francisco, CA; ∥ Department of Gastroenterology and Hepatology, Seattle Children’s Hospital, Seattle WA; ¶ Division of Gastroenterology, Department of Pediatrics, University of Washington, Seattle, WA.

**Keywords:** inflammatory bowel disease, dietary therapy, qualitative studies

## Abstract

Because of the high cost and associated toxicities of pharmacotherapy treatment for inflammatory bowel disease (IBD), there has been growing interest in dietary therapy. The objective of this study is to assess barriers to initiating or maintaining the specific carbohydrate diet (SCD) to inform strategies for improving access and adherence to the diet.

**Methods:**

We conducted semistructured interviews with parents of 10 children with IBD receiving care at a single academic treatment center. Parents were eligible if their child with IBD was either currently on the SCD, previously on the SCD, or opted not to initiate the SCD. Core questions were developed in conjunction with IBD clinical experts. Interviews were transcribed and analyzed using an inductive approach.

**Results:**

Parents of children diagnosed with IBD primarily chose to try the SCD because of concerns about medication safety. Three major barriers to utilizing the SCD emerged: cost, time commitment, and psychosocial impact. Many parents also expressed that following the SCD got easier over time and some parents experienced spillover effects of improved personal health and understanding of nutrition. All parents were strong proponents of the importance of diet in managing IBD and expressed desire for more research into the SCD and other forms of dietary therapy.

**Conclusions:**

These findings provide important insight into factors affecting utilization of the SCD in pediatric IBD. Further research is needed to develop interventions or strategies to diminish these barriers and enable more patients to benefit from the SCD.

**What is Known**

- Inflammatory bowel disease (IBD) confers a substantial economic burden on families and healthcare systems, and medications are the leading driver of direct costs among patients with IBD.
- Given the high cost of pharmacotherapy and risk of adverse effects, considerable attention has been given to dietary therapy as a potential alternative or a supplement to pharmacotherapy for IBD.
- Multiple observational studies show the specific carbohydrate diet (SCD) provides symptom relief and improves inflammatory burden and mucosal healing in pediatric patients.

**What is New**

- To our knowledge, this is the first study examining barriers to the SCD among pediatric IBD patients.
- Parents of children diagnosed with IBD primarily chose to try the SCD because of concerns about medication safety.
- The three major barriers to utilizing the SCD were cost, time commitment, and psychosocial impact.
- Parents were strong proponents of the role of diet in IBD but several felt more research is needed.

## INTRODUCTION

In North America, more than 1.3% of the population has inflammatory bowel disease (IBD), amounting to over 3.1 million Americans (1). A quarter of new diagnoses occur during childhood with incidence of pediatric IBD increasing globally over the last half century (2). Pediatric IBD is particularly aggressive, leading to growth failure, high surgery rates to manage pharmacotherapy-resistant disease, and decreased quality of life (3,4). In addition, IBD confers a substantial economic burden on families and healthcare systems (5,6). In a recent study of pediatric IBD patients, mean 5-year costs of care were $45,753 for Crohn’s disease (CD) and $50,516 for ulcerative colitis (UC) (7). Medications are the leading driver of direct costs among IBD patients, accounting for ~35% of total costs (8–10), greatly outpacing hospitalization and surgery costs.

Current therapies for IBD focus on medications that suppress the immune system’s ability to instigate and perpetuate an inflammatory reaction in the gastrointestinal tract. Choice of therapy is determined by disease severity, IBD-related complications, prior medication failures, and need for surgery (9). Treatment approaches for IBD may follow a step-up approach, beginning with therapies such as corticosteroids and immunomodulators (eg, methotrexate, 6-mercaptopurine, azathioprine) (10), or begin with biologic therapies (11), such as tumor necrosis factor (TNF) antagonists. Although these therapies are effective for many, not all patients respond. For example, with Infliximab, a commonly prescribed biologic, up to 30% of patients do not respond, and almost 50% of responders lose clinical benefits within 1 year (12,13). Additionally, anti-TNF treatment is associated with multiple safety concerns, such as opportunistic infections, malignancies, and adverse drug reactions (14).

Given the high cost of pharmacotherapy and risk of adverse effects, considerable attention has been given to dietary therapy as a potential alternative or a supplement to pharmacotherapy for IBD. The specific carbohydrate diet (SCD) is a dietary program created by Dr. Sidney Haas in the 1920s (15), and popularized by Elaine Gottschall with her book, *Breaking the Vicious Cycle: Intestinal Health Through Diet* (16). Dietary intervention is based upon the role of intestinal dysbiosis as a presumed primary immunologic trigger in IBD. With diet a major determinant of intestinal microbiome composition, diet change has been shown to effectively and reproducibly alter the microbiome (16). The goal of the SCD is to eliminate factors contributing to dysbiosis, supporting an eubiotic microbiome (17).

Multiple observational, uncontrolled studies show the SCD provides symptom relief, improves inflammatory burden, and improves mucosal healing in pediatric patients (18–21). A survey of patients on the SCD found most patients perceive it to construe clinical benefit (22). More recently, the PRODUCE study, a large multicenter prospective cohort study confirmed the anti-inflammatory effect of the SCD (23). Although not specific to the SCD, many physicians indicate a lack of comfort with providing nutritional counseling (24–26). As evidence of the benefits of dietary interventions such as the SCD continues to grow, it is imperative for physicians and parents to understand factors affecting utilization. Thus, we conducted a qualitative study of parents of IBD patients treated at a single clinical institution to assess barriers to initiating and/or maintaining the SCD to inform strategies for improving access and adherence to the diet.

## METHODS

### Recruitment

Participants were enrolled from the IBD Center at Seattle Children’s Hospital, a clinic providing comprehensive multidisciplinary treatment for approximately 300 IBD patients annually. Recruitment was conducted via purposive sampling (27) to identify parents of patients who met predefined criteria. Parents were eligible if their child with IBD was either currently on the SCD, previously on the SCD, or opted not to initiate the SCD. Parents were contacted via phone and asked if they were interested in participating in the study. If they agreed, they were sent an online consent form and a HIPAA-compliant Zoom interview was scheduled. Interviews were conducted until the point of saturation (28,29)—the point at which no new themes emerge from data collection. Evidence suggests saturation can occur within 10 interviews when the research scope is narrow (29). All study procedures were approved by Institutional Review Boards at University of Washington (00006878) and Seattle Children’s Hospital (00002774).

### Interviews

Interviews were semistructured. Core questions were developed in conjunction with IBD clinical experts. Interviews also explored considerations raised during the course of conversation. For parents of patients currently on the SCD (n = 4 patients), questions addressed reasons for choosing to use the SCD, satisfaction, as well as shopping, eating, and food preparation routines before/during the SCD. Parents of patients with past SCD use (n = 4 patients) were also asked about reasons for discontinuation. Parents of patients who did not initiate the SCD (n = 2 patients) were asked about reasons for not pursuing it as a treatment option.

### Qualitative Analysis

Interview transcripts were imported into Taguette (30) and coded using an inductive approach (31). The lead coder (NRMS) performed a close reading of the text, developing categories that encapsulated prominent themes. NRMS then completed an iterative process of revising and refining categories to create a final codebook that was used by both coders (NRMS and SM) to independently code responses. After coding was complete, the coders compared their coding for consistency.

## RESULTS

### Participant Demographics

We conducted a total of 9 interviews with 12 parents of 10 patients (two patients were siblings). All contacted parents chose to participate. Two patients never initiated the SCD, four were previously on the SCD, and four were currently on the SCD. Among patients currently on the SCD, three were using it as the only therapy, whereas one was also using pharmacotherapy (Table 1).

**TABLE 1.** Characteristics of Pediatric Patients With Inflammatory Bowel Disease Whose Parents Participated in Qualitative Interviews About the Specific Carbohydrate Diet

	SCD Initiators (n = 8)		Non SCD Initiators (n = 2)
	Current Users (n = 4)	Past Users (n = 4)	
Sex, n (%)			
Male	3 (75%)	1 (25%)	2 (100%)
Age (yrs), median (range)	13.5 (11–15)	15.5 (13–18)	15.5 (13–18)
Duration of disease (yrs), median (range)	3.5 (0.5–4)	1.5 (0.6–6)	6 (3–9)
Disease type, n (%)			
Crohn’s disease	2 (50%)	1 (25%)	1 (50%)
Ulcerative colitis	2 (50%)	3 (75%)	
Indeterminate colitis			1 (50%)
Current therapy, n (%)			
SCD only	3 (75%)		
SCD plus pharmacotherapy	1 (25%)		
Pharmacotherapy only		4 (100%)	2 (100%)

SCD = specific carbohydrate diet.

### Family Involvement

Among the nine families interviewed, one patient followed the SCD alone, two sets of parents followed the SCD with the patient, and another parent chose to follow the SCD with her daughter, whereas other family members did not. The remaining families (n = 4) had SCD-friendly dinners, but family members did not adhere to the SCD for other meals.

### Decision Process

The primary rationale for SCD initiation was concern about the use of pharmacotherapy. Parents (n = 8) expressed desire to try the SCD initially because of concerns about medication toxicity and long-term adverse drug effects. One parent also noted concerns about the child’s future health insurance and affordability of medications, saying, “it seems more sustainable than long term drugs, I know a lot of the drugs are lifetime and not sure how well off [he] will be… financially, so affording those drugs, or getting health insurance when he turns 18 might be a struggle.”

Two patients were noninitiators. One child had too many food allergies for it to be feasible. Allergies to ingredients like nuts, commonly used on the SCD, would have made it harder to follow and further restricted his diet, interfering with adequate nutrition. The other child never initiated the SCD because his disease was too severe. At diagnosis, the patient was significantly underweight. The priority was supplying him with adequate calories; restricting his diet may have interfered.

Four SCD initiators later discontinued the diet. One patient took a trip abroad where he was unable to adhere to the diet and acquired a *C. difficile* infection that required him to initiate pharmacotherapy, at which point he chose not to continue the SCD. The other three discontinued because of lack of response.

### Potential Barriers

#### Cost

Most (n = 7) parents of SCD initiators reported increased grocery expenses. For one family, cost presented enough of a hardship they stopped following the SCD with their daughter. Other parents could afford the extra expense, but recognized cost would likely be a barrier for lower-income families. Primary drivers of increased grocery costs were organic produce and specialty ingredients. Specifically, many SCD recipes include nut flours or milks, which can be double the cost of wheat flour and dairy milk.

Parents utilized a variety of strategies to reduce financial burden such as buying SCD ingredients in bulk (n = 6). Additionally, although organic foods are perceived by many parents to be healthier, they are not required, and one mother was able to save money by purchasing a mix of conventional and organic produce. As a longer-term solution, one parent suggested, “maybe there would be a way… you could submit some of your grocery receipts for eligible items for Flexible Spending Account reimbursement, or your insurance, or some kind of financial help to help people make it feasible for them.”

#### Time Commitment

Parents of all SCD initiators, even those who cooked frequently before the SCD, spent more time planning and preparing meals. One factor contributing to this was the need to invest more time in food preparation, because many premade items (eg, broths, sauces) contain ingredients not allowed by the SCD. Parents of two patients specifically noted increases in time spent baking and three parents noted lunches and snacks were challenging. One parent described, “the toughest transition was figuring out what I could send for lunch, because before he was diagnosed we just always got him a school lunch.” Additionally, two patients’ parents needed to shop more frequently, and at a wider variety of stores, because of the need for more fresh produce and special ingredients. Parents of six patients spent more time in stores because they had to read product labels closely to ensure ingredients were allowed by the SCD.

Parents utilized Facebook groups (n = 6), online resources (n = 3), and cookbooks (n = 2) for recipe ideas. One parent reported, “I just found all these websites and people that are doing SCD and just started following them and finding recipes.” Another parent found success “keep[ing] things really simple in the beginning.” She advised parents not to “fixate on trying to recreate their child’s favorite foods.” Other parents (n = 3) found it easier to substitute ingredients (eg, adding more vegetables to soup in place of rice) than to design new meal plans.

One solution for the need to visit multiple stores is that mainstream grocery stores are starting to carry more SCD-legal foods. One parent said, “these days it seems easier to find it locally then to have to drive to those specific stores.” Ordering ingredients online also helped reduce time spent grocery shopping.

#### Psychosocial Impact

Four patients’ parents spoke about psychosocial impacts of the SCD on their kids. For some patients, the SCD was difficult because they missed specific foods. For example, one child “was quite unhappy that she couldn’t eat bread.” Other children felt left out in social situations when food was present.

Two parents highlighted the role of social support to lessen the psychosocial difficulty of following the SCD. One patient’s extended family makes an effort to cook things he can eat when he visits. His mother said, “It’s really nice to have that level of support.” Another patient’s siblings, who do not follow the SCD, each “had a recipe that they would make for her.” This helps the patient feel supported by her family even if she cannot eat the same foods they eat.

One parent whose child was initially opposed to the SCD spoke about the importance of the child being on board. She did not think it would have been successful had she forced her daughter to follow the diet. She shared her strategy for helping her daughter cope with the psychosocial difficulties, saying “I want to be realistic, but also frame it in a positive light… we’re putting stuff in that is healing and that we’re eating real foods. And so rather than feeling like completely left out, I have also tried to talk about wow, we’re so lucky and we’re kind of privileged that we get to eat these real foods that are not as processed.” This parent and another recounted success bringing their own food to restaurants or social gatherings. One patient’s parents called restaurants ahead of time to inquire about ingredients and often found that menu options that could easily be adjusted to be SCD-friendly.

### Additional Themes

#### Adapting to the SCD

All parents expressed that while the SCD initially seemed overwhelming, it gets easier over time. One parent described, “you just get used to the new way of doing stuff.” Initially, the diet involves a lot of experimentation and learning in the kitchen, but “it’s not nearly as overwhelming after you get it figured out and get the base ingredients down.” Once parents identified recipes and successfully created dishes the child enjoyed, preparing and planning meals became less time-consuming and burdensome.

#### Spillover Effects

Parents of six children said the SCD improved their own health and understanding of nutrition. Specifically, reading labels led parents to make dietary changes for themselves as well as for their child. One father following the SCD with his son lost weight and felt his digestion improved.

#### Desire for More Information

Parents of five patients expressed desire for more research into the SCD and other dietary therapy. The mother whose son did not use it because of severe disease at diagnosis said, “if it worked for him, I would choose that, in a heartbeat over the bucket of medication that he’s getting today… I would love … if your research shows that… it does work on someone that’s got his kind of case.” She wanted to know if there was a way to “phase into” the SCD for patients using pharmacotherapy to avoid risking a scary and perhaps dangerous disease flare.

Parents of two siblings who discontinued the SCD also wanted more data. The father said, “what works, and how to kind of space it with other treatments and how to monitor it etc., it’s far from clear.” The mother added, “the hardest part is following something that you’re not sure if it is proven.” Similarly, the mother of the child who did not initiate the SCD because of food allergies is hopeful for research into dietary therapy options better suited to children with food allergies. A parent whose son had success with the SCD underscored how important research is to establish the SCD as a “legitimate treatment.” She explained, “having published data out there is hugely important for the success of, and a widespread knowledge of, the diet.” These parents are strong proponents of the role of diet in IBD but feel more research is needed.

## DISCUSSION

With knowledge of clinical and anti-inflammatory impacts of the SCD in IBD continuing to grow, understanding barriers to initiating and maintaining dietary therapy is essential to achieving better patient outcomes. In our study, parents of children diagnosed with IBD primarily chose to try the SCD because of concerns about medication safety. This finding is in accordance with a 2015 case series of 50 adult IBD patients that reported 82% (n = 41) of patients opted to follow the SCD caused by fear of long-term medication consequences.

Three major barriers to utilizing the SCD emerged from the interviews: cost, time commitment, and psychosocial impact. Lack of research on this topic makes it difficult to compare to prior studies, however, evidence from other populations supports the plausibility of these findings. For example, research shows processed foods, such as those not permitted by the SCD, are less expensive than their unprocessed alternatives (32). Additionally, a cross-sectional population-based survey from 2008 to 2009 found more frequent intake of vegetables and fruits, and lower intake of convenience foods were associated with greater amount of time spent preparing food at home (33). In adult IBD patients, diets that restrict grains/carbohydrates are associated with decreased food-related quality of life compared to either no dietary therapy or other dietary therapies (mean Food-Related Quality of Life [FRQol-29] score 71.0 versus 84.8 [scale 29-145, higher = better], *P* = 0.02) (34).

Multiple policy changes could facilitate wider-spread adoption of dietary interventions such as the SCD and lessen the impact of the barriers we identified. Incorporation of dietary therapies into clinical guidelines from medical entities like the American Gastroenterological Association would enhance clinical knowledge of the diet’s therapeutic potential. An “SCD-legal” label from the Food and Drug Administration might increase awareness and encourage more SCD-friendly options at school cafeterias and workplaces (in addition to options for inpatient food services at hospitals), similar to the impact of the FDA’s “gluten free” label (35). This would also make it easier for patients to quickly identify safe items in grocery stores. Additionally, many countries either offer free gluten-free staples or subsidies to patients with celiac disease (36,37). In the United States, patients with celiac disease can deduct costs of gluten-free foods from their taxes or use Flexible Spending Account provisions to pay for the incremental cost of foods, including shipping expenses (37). A policy similar to this for the SCD would help diminish any economic barrier for patients, thereby improving adherence and leading to improved health outcomes.

### Limitations

Much of the burden associated with the SCD (ie, cost and food preparation time) falls on parents. Many IBD patients are still at an age at which they rely on parents for food, and parents tend to shoulder the burden of transporting children to and from infusion, and managing oral medication administration (38). However, although interviewing parents provides important insight into logistical and practical aspects of adopting the SCD as a treatment option, it does not allow for direct assessment of the patient perspective. This is an important consideration and is an area that should be explored in future research.

The patient population served by Seattle Children’s Hospital is not representative of the national population of pediatric IBD patients and parents. Thus, these results may not be generalizable to the broader population of families with children with IBD. Specifically, there is significant national geographic variation in affordability and access to fruits and vegetables in the United States (39), so some of the potential barriers identified in this study may be more salient for families in other parts of the country.

Additionally, it is important to note that although the goal of this study was to highlight barriers and facilitators to adopting and maintaining the SCD rather than predictors of effectiveness, clinical effectiveness is certainly a facilitator.

## CONCLUSION

To our knowledge, this is the first study examining barriers to the SCD among pediatric IBD patients. Our results suggest cost, time commitment, and psychosocial factors are the primary concerns impacting patients’ ability to adhere to the diet. Further research is needed to develop interventions or strategies to diminish these barriers and enable more patients to benefit from the SCD.

**TABLE 2.** Potential Barriers to Utilization of the Specific Carbohydrate Diet Among Parents of Pediatric Patients With Inflammatory Bowel Disease and Example Quotes From Qualitative Interviews

Potential Barrier	Example Quotes
Cost	You know, but a barrier, and this is, this is the part that breaks my heart a little bit, and I, is that the barrier for some families is the cost. Not only, I mean yes, there’s a time investment, but there’s also just the cost of good quality produce and good quality food is higher.” It just got to a point when I was just like ok, um, I don’t think we can do this, all of us do this diet because it was just getting too expensive. I could see how a low income family might struggle with it. Especially at first you’re just buying a lot of the whole foods aren’t cheap. It definitely got more expensive because of just trying to buy all the good fruits and vegetables I think was the main problem…and I forget the name of the bread, but it was made out of cashews, and buying organic cashews were expensive, and that bread was like three cups of cashews for each loaf, and I was just buying these bags from amazon and it was like $50 each, and also like making her almond milk and buying good almonds to make the almond milk, and then also like, I would just try to like, I was just trying to find her good snacks that she could enjoy that I wasn’t preparing all the time, that she could just grab from the pantry and go, and just purchasing um some of the SCD stuff that they were selling online, those were very pricey. Because all those nut flours and milks that are not cow milk, they are very expensive. We’re lucky ‘cause we can spend the money on the groceries and stuff. It’d be interesting to see if you had a low income family where generally you’re going to buy cheaper, more processed stuff. I think it’s the organic, like the organic vegetables. I think that’s it, and then when we order stuff online, it is so much money to have stuff shipped to us, especially if it needs to be in a cooler or something, so that costs a lot, so we don’t do that too often. But yeah I think it’s the organic. And then I think it’s the organic vegetables like organic fruit that costs a lot.
Time commitment	We used to do a lot of premixes, canned goods, that kind of stuff that we just throw together. Now everything’s from scratch, so, it’s an hour or two depending on the meal. Quite a bit more baking with those special recipes for him and it mainly just takes time to figure them out rather than to make them. I mean some of them are harder to bake, but finding them and then you know, there are a lot of flour, or almond flour based breads that turn out horrible, and then there’s the ones she’s recently made are really good. I go twice a week now, I don’t find that produce lasts long enough to go only once a week. And his diet is a lot of produce. There are items that we find that work for us, and then the store will stop carrying it, so I’ll have to shop around to try to find it somewhere else. It made me have to go to more stores. Because not one, there was no one store that carried everything.
Psychosocial impact	I think it was, it was also hard, because he was in a, you know it’s a sensitive part of your life when from uh, you’re going from uh 12, well you know, that’s tough. I mean it’s almost easier when you’re little. Right. And then if you’re a young adult I think it’s easier. But when you’re right in the middle it’s hard. So I think it was challenging for him when he was in school and stuff. It’s emotionally difficult for ***. When he goes to school and there’s a birthday party, and everybody else has cupcakes and he can’t have that. Um. He’s been great with it, but it’s difficult for him to not be able to eat some of the things that even siblings are able to eat, but he’s not. When I read the diet, I’ll be honest, my stomach turned and I felt like I was going to throw up because I was like this is everything he loves. This kid ate rice like crazy. He ate, he loved, he would guzzle milk. I mean, it was like, he’s gonna lose everything he loves and I was like oh my gosh.

## ACKNOWLEDGMENTS

We would like to thank Meghan Schneider for transcribing the interviews.
